# Sub-wavelength terahertz imaging through optical rectification

**DOI:** 10.1038/s41598-018-31970-w

**Published:** 2018-09-10

**Authors:** Federico Sanjuan, Gwenaël Gaborit, Jean-Louis Coutaz

**Affiliations:** 1 IMEP-LAHC, UMR CNRS 5130, University Savoie Mont-Blanc, 73376 Le Bourget du Lac Cedex, France; 2 Kapteos, 354 voie Magellan, 73800 Sainte-Hélène du Lac, France

## Abstract

We record a sub-wavelength terahertz image of a caster sugar grain thanks to optical rectification in the sample excited with a femtosecond laser beam. The lateral spatial resolution of this technique is given by the laser spot size at the sample and here its measured value is 50 *μ*m, i.e. ~λ/12. We give an estimation of the ultimate resolution that could be achieved with this method.

## Introduction

Production of images in the terahertz (THz) spectral range with a sub-wavelength resolution is nowadays commonly achieved through near-field techniques^1,2^. Most of the setups employ time-domain methods with femtosecond laser excitation of the THz emitting devices, but images have also been obtained with continuous THz sources like QCL^3^ or even with synchrotron radiation^4^, allowing the study of biological tissues^5^. Such techniques are extremely efficient and permit to observe nanometer details^6,7^. On the other hand, they require dedicated instrumentation and know-how, which are not available in every laboratory.

Another solution is to generate, by optical means, the THz signal at the surface of the studied sample. This has been greatly demonstrated for years^8,9^ by Japanese teams, who have produced THz pulses by exciting electronic integrated circuits with a femtosecond laser. Carriers are photo-excited in the semiconductor substrate by the laser pulses and then are accelerated by any electric field along interconnections and components of the integrated circuit. These accelerated free carriers radiate a THz signal, which allows one to map the electric field at the studied device and for example, to detect any failure in the electronic circuit. Here the spatial resolution of this mapping is limited either by the distance over which carriers accelerate or by the laser spot size. Typically this technique, called laser THz emission microscopy (LTEM), exhibits a micron spatial resolution^7^. It allows also evaluating the surface state of semiconductors^10,11^ or the electrical properties of semiconductor components^12^. A related technique was employed to produce THz sub-wavelength images by Lecaque *et al*.^13^. The sample is put on the surface of a nonlinear crystal. The laser pump beam is focused in the nonlinear crystal in which it produces a THz signal by optical rectification (OR) and the OR-generated THz pulse goes through the sample before reaching the receiver. Thus the recorded THz intensity is modulated by the near-field transmittance of the sample. The image is obtained by scanning the laser beam over the sample and a *λ*/10 (30 *μ*m) lateral resolution is demonstrated after a numerical deconvolution of the image. A method inverse to that of Lecaque *et al*.^13^ was published by Blanchard *et al*.^14^: here the sample is put over an electro-optic crystal that is illuminated by a wide THz beam. The THz field, which is scattered and diffracted by the sample, is read by the probe laser beam that is tightly focused at the surface of the crystal. Thus, the THz transmission of the sample is measured in the near field and the authors report a *λ*/30 (14 *μ*m) lateral resolution. Thanks to a very powerful THz beam generated by tilted-pulse-front excitation in LiNbO_3_, images of 370 × 740 *μ*m^2^ samples are captured at a rate of 35 frames per second.

These two techniques, i.e. photo-carriers acceleration in semiconductors and nonlinear effects in crystals, differ notably because, in the first one, the sample serves as a source of THz radiation, while in the second one, the sample perturbs the emission or detection of THz waves in a nonlinear crystal. Here we propose to benefit from the advantages of both techniques, i.e. to employ optical rectification (OR) through or at the surface of the sample in view of getting a THz image with a sub-wavelength resolution. Thus the technique is not limited to semiconductor devices and it can be applied to the characterization of dielectric samples, as soon as they show a nonlinear optical response. As compared to the techniques by Lecaque *et al*.^13^ or by Blanchard *et al*.^14^, which deliver a signal proportional to the transmission of the sample at THz frequencies, here the recorded signal depends on both the sample absorption at visible and THz frequencies and on sample crystallinity through the nonlinear tensor. Indeed, when the sample material is not centro-symmetric, focusing a powerful beam at its surface induces second order optical nonlinear phenomena in the enlightened region, i.e. second harmonic generation (SHG) and OR. The SHG technique is widely spread in laboratory and hospitals and it delivers superb images that render for the sample crystalline inhomogeneity^15^. For example, membranes of biological cells are highly symmetrical due to the preferred orientation of their molecules and thus membranes produce a rather strong SHG signal that also depend on the membrane potential^16^, while some inner parts of the cells are liquid and thus are almost amorphous, leading to no SHG signal or at least to a weak one. Thus the proposed method is complementary to SHG imaging, since a THz photon is produced any time a SHG photon is. However, the efficiencies of OR and SHG are different, as recorded signals depend not only on the nonlinear tensor, but also on the susceptibility of the material respectively in the THz and visible ranges. Therefore, the two techniques should bring complementary information on the sample. It should be also noted that, even if the number of generated THz and SHG photons at the molecular level are the same and even without taking into account propagation and absorption effects, the THz beam should be weaker than the SHG one, because the energy of THz photons is about 1000 smaller than those of visible ones. In fact, OR was already used to study biological materials (bacteriorhodopsin)^17^: the intense impulse polarization excitation leads to a strong resonant infrared (IR) radiation from the molecules. Such IR light, whose wavelength is about 10 *μm*, is easier to detect than the THz one, because the IR photons are about 25 times more energetic than the THz ones. However, this result by Groma and colleagues^17^ demonstrates that OR could deliver information on the molecular response of the samples, even if the imaging purpose was not addressed in their work.

In this paper, we demonstrate the proof of concept of this technique by recording for first time a THz–OR image of a grain of caster sugar, taken from a packet of caster cane sugar (brown sugar) bought in a grocery. Sugar presents the advantage of being an efficient and very cheap nonlinear material, which has already been used for SHG^18–21^. Moreover, caster cane sugar grain are highly crystalline, with almost cleaved faces, even if they are not single crystals and contain impurities. Furthermore, we give the ultimate performance in terms of spatial resolution of this technique.

To our knowledge, the idea of THz imaging through OR was only published by a Japanese team^22–24^, in view of observing ferroelectric domains and especially domain walls in supramolecular ferroelectrics. Impressive images were obtained, but microscopy was not the main goal of this study. As compared to their work, we address here for first time any kind of material with the aim of mapping the crystalline structure of the sample through its nonlinearity and we discuss about the limitations (especially the spatial resolution) of this technique.

## Results

**Figure 1:**
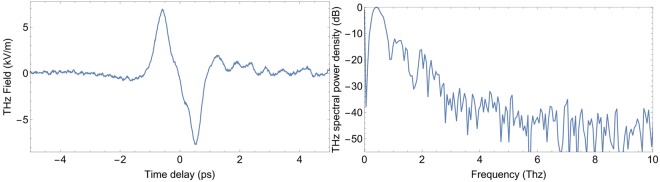
THz waveform (left) and corresponding power spectrum (right) generated by OR in a sugar grain.

Figure 1 shows a typical THz waveform and its spectrum obtained with a rather big grain (thickness ~0.8 mm). The mean laser power is ${\overline{P}}_{laser}\,=\,$3.65 mW (3.65 *μ*J per pulse), corresponding to a peak power density ${D}_{laser} \sim 2.8\times {10}^{12}$ W/cm^2^. The spectrum is maximum around 0.495 THz and spreads up to 5~6 THz where it reaches the noise level (about −50 dB). Some strong dips are seen around 0.9 and 1.7 THz. As sugar does not present absorption peaks at these frequencies, they may be due to water vapor absorption lines^25,26^ or to tiny rebounds of the laser pulse in the rubber tape where the sugar grain was stick.

Let us notice that the sample is almost transparent at the pumping laser wavelength and that we didn’t observe any two-photon absorption effect: therefore THz emission by photo-generated carriers can be ignored. However, to definitively demonstrate that the observed THz signal is originating from OR, we performed measurements of the polarization state of the generated THz beam. As expected, we found an angle dependency between the relative positions of the sugar crystal with regards to the pump laser polarization angle, which is the signature of OR.

**Figure 2:**
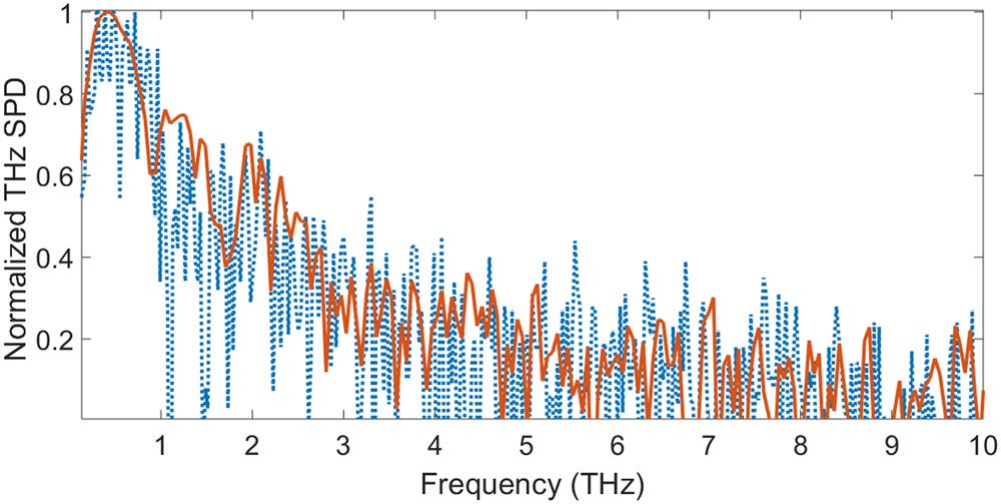
Normalized power spectra generated by OR in a sugar grain (red continuous line ${D}_{laser} \sim 2.8\times {10}^{12}$ W/cm^2^, blue dashed line ${D}_{laser} \sim 43\times {10}^{10}$ W/cm^2^). Both spectra are normalized to a dynamic range equal to 1.

The recorded spectra obtained with grains of different thicknesses or for different pump powers present similar results. This is shown in Fig. 2 where the THz power spectral density (log scale, with a dynamic range normalized to 1), is plotted for the largest and weakest recorded signals, generated with ${\overline{P}}_{laser}=3.65$ mW (${D}_{laser} \sim 2.8\times {10}^{12}$ W/cm^2^) and ${\overline{P}}_{laser}=0.056$ mW (${D}_{laser} \sim 0.043\times {10}^{12}$ W/cm^2^) respectively.

The weakest THz pulse energy is thus about 4200 smaller than the largest one. The spectra are rather similar, but the weakest one is of course noisier.

**Figure 3:**
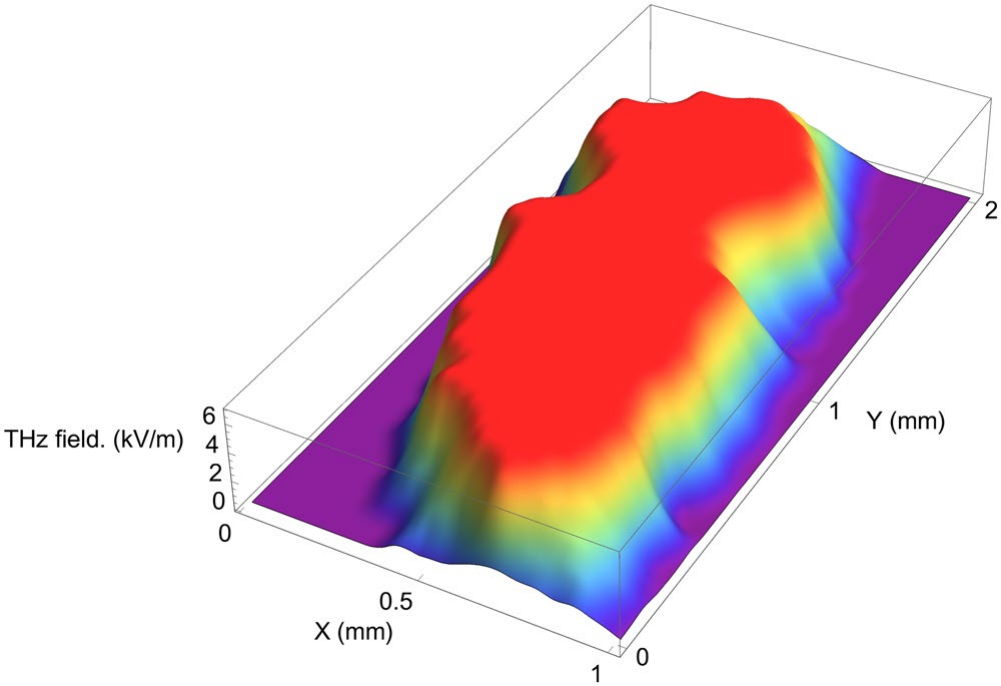
3-D surface plot of the recorded THz OR image.

This allows us to record only the peak amplitude of the THz waveforms, which makes the image recording time much shorter. Therefore, the effective experimental frequency is the one of the maximum of the THz spectrum, *i.e*. ~0.5 THz. The OR-THz image of a sugar grain is depicted on Fig. 3. Points are recorded every 10 *μ*m in both directions. At the center of the grain, the OR-THz signal magnitude is almost constant, while it decreases strongly at the crystal border.

**Figure 4:**
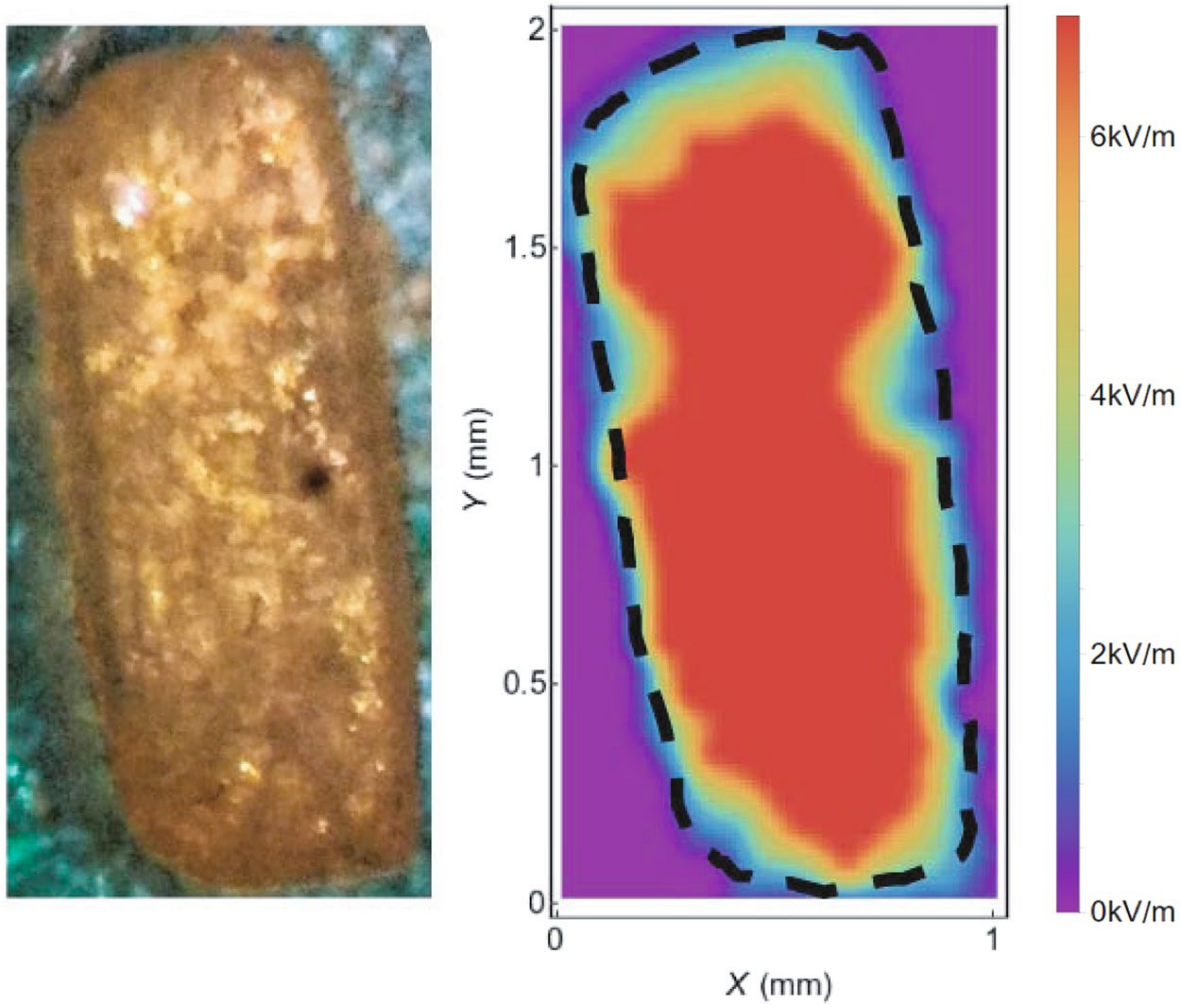
Image (left side): Photography of the studied caster sugar grain. Image (right side): THz OR image. The border of the grain sugar deduced from the photography, is shown as a black dashed line.

Figure 4 presents a photography of the grain (left) and the superposition of the sugar crystal outline from photography (black dashed line) with the OR-THz 2D plot extracted from Fig. 3 (right). The THz signal is generated only by the sugar grain and thus the OR-THz map resembles the grain shape. However, the OR-THz image reveals details at the crystal border that are not seen on the photography and that could be attributed to the sides of the grain that are not sharp or vertical, for example because of slivers. Moreover, nearby the top of the sample, the decrease of the THz signal does not follow the sample border: this could be due to impurities, to a lack of crystallinity or to the presence of water at these locations. Since we are making an imaging proof-of-concept, we don’t go further in this analysis. However, this new information about the crystal could be useful to test its quality.

**Figure 5:**
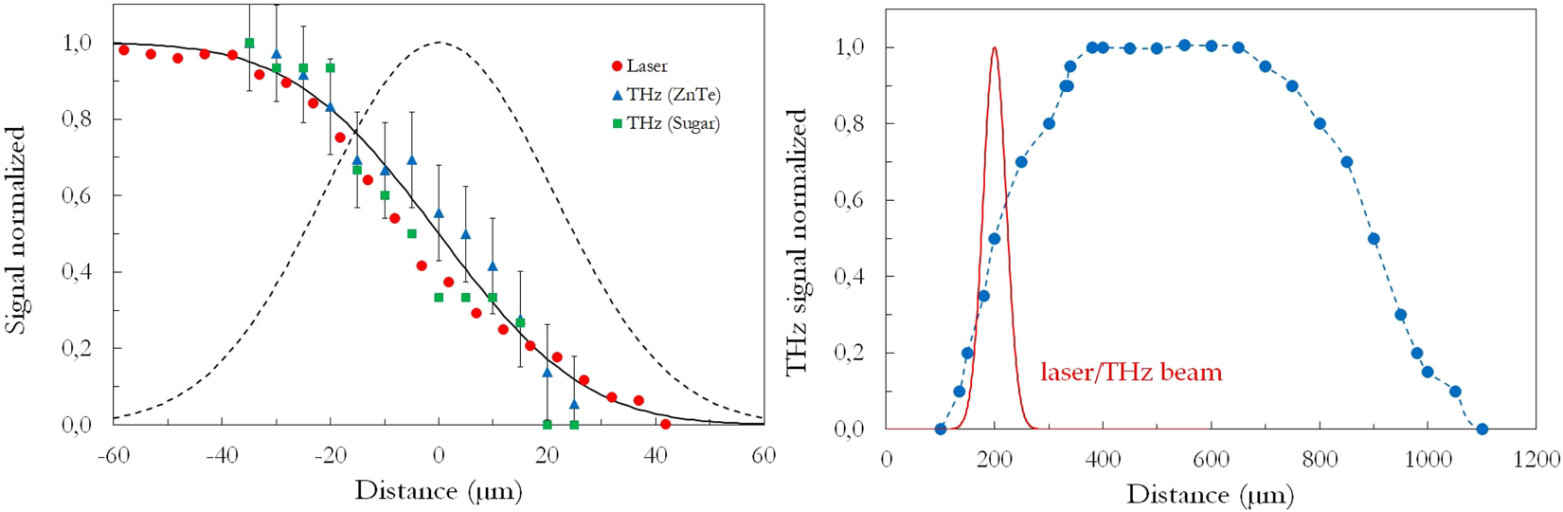
Left: Razor blade signal of the laser beam (red circles) and of the OR THz beam, either generated by a sugar grain (green squares) or by a ZnTe wafer (blue triangles), together with a complementary error function fit (continuous line, *w*_*laser*_ = *w*_*THz*_ = 30 *μ*m) (the corresponding Gaussian profile is plotted as a dashed line); Right: OR THz signal profile versus the distance along the sugar grain (blue circles and dashed line). The continuous red line represents the laser or THz beam profile at the sample, given by the razor blade experiment.

The decrease of the THz signal at the grain border depends on the shape of the grain side, but also on the waist of the laser beam and on the diffraction of both laser and THz beams by the sample edge and thus it reveals the lateral spatial resolution of the measurement. To determine this spatial resolution, we have measured the waist of the laser beam at the grain surface location using a razor blade technique. The measured data (Fig. 5 left) are well fitted with a complementary error function, which attests that the laser beam is Gaussian and permits us to determine the laser beam waist (radius) equal to *w*_*laser*_ = 30 *μ*m (the Gaussian shape of the laser beam is plotted as a dashed line in Fig. 5 left). We have also applied the razor blade technique to the generated THz signal. The data are almost superimposed with the laser ones, *i.e. w*_*laser*_ = *w*_*THz*_ which proves that the THz signal is only generated by the illuminated part of the sugar grain and that the THz beam is also Gaussian. This is confirmed by similar data (see Fig. 5 left) recorded using a perfectly flat ZnTe wafer, for which scattering and diffraction effects do not perturb the THz beam shape. Therefore, following the Rayleigh criterion, the lateral resolution of our record is ${\delta }_{THz}=2{w}_{THz}\sqrt{{\rm{l}}{\rm{n}}\mathrm{(2)}}=50$ *μ*m.

Figure 5 right depicts the profile of the OR-THz signal versus the position along a line that crosses the grain s(a horizontal line roughly at the center of the grain shown in Fig. 5). As already seen in Fig. 3, the THz signal is practically constant at the center of the grain, which reveals that the grain thickness as well as the grain homogeneity are also regular. The decrease of the OR signal at the grain border occurs over a distance of 300~400 *μ*m. This is not due to the spatial resolution of the experiment, since both laser and THz beams are much narrower (see continuous red line on Fig. 5 right): this is validated by performing the deconvolution of the OR profile, which does not show noticeable differences with the profile plotted here. This smooth decrease is thus attributed to the shape of the sugar grain and thus to a reduction of the grain thickness nearby its borders.

## Discussion

Let us evaluate the limit of the spatial resolution in the OR imaging technique. We suppose that OR occurs at the surface of the sample and thus phase-matching effect may be neglected (in the present case, the sample thickness is more or less equal to the THz wavelengths, which justifies that propagation effects -phase-matching- may be ignored). As well, because we only derive order of magnitude, thus we forget about the tensorial behavior of the nonlinear phenomenon (the nonlinear susceptibility tensor $\overleftrightarrow{\chi }$ is simply written as a scalar *χ*). It follows that the magnitude *E*_*THz*_ of the THz field is proportional to the pump laser field power *P*_*laser*_:

1 $${E}_{THz}\propto |\overleftrightarrow{\chi }\,:{\overrightarrow{E}}_{laser}\cdot {\overrightarrow{E}}_{laser}|\propto \chi {E}_{laser}^{2}\propto \chi \frac{{P}_{laser}}{{S}_{laser}}.$$

Here *E*_*laser*_ is the laser field magnitude (note that we use here only peak values of either the laser or THz pulses and not averaged values) and ${S}_{laser}\propto {w}_{laser}^{2}$ is the laser spot surface at the sample surface. The radiated THz power *P*_*THz*_ is given by:

2 $${P}_{THz}\propto {S}_{THz}{E}_{THz}^{2}\propto {S}_{laser}{E}_{THz}^{2}\propto {\chi }^{2}\frac{{P}_{laser}^{2}}{{S}_{laser}}.$$

To derive this expression, we state that, at the sample surface, the THz spot size is the same as the laser one, i.e. *S*_*laser*_ = *S*_*THz*_ since the THz signal is generated by the illuminated area of the sample. Because this illuminated area is much smaller than the THz wavelength, the radiated THz field is almost a spherical wave. This wave is collected by a lens of section *S*_*lens*_ located at distance *r* from the sample. The collected THz is then focused onto the receiver. The THz power *P*_*THz*,*d*_ impinging the receiver is:

3 $${P}_{THz,d}\propto {S}_{lens}\frac{{P}_{THz}}{{r}^{2}}\propto {\chi }^{2}\frac{{S}_{lens}}{{r}^{2}}\frac{{P}_{laser}^{2}}{{S}_{laser}}\propto {\chi }^{2}{{\rm{\Omega }}}_{lens}{S}_{laser}{D}_{laser}^{2}.$$

Ω_*lens*_ is the solid angle subtended at the sample source by the entrance aperture of the THz optical system and *D*_*laser*_ is the laser power density at the sample surface. It follows that the laser waist *w*_*laser*_ at the sample can be expressed as a function of *P*_*THz*,*d*_, the nonlinearity *χ* and *D*_*laser*_:

4 $${w}_{laser}\propto \sqrt{\frac{{P}_{THz,d}}{{{\rm{\Omega }}}_{lens}}}\frac{1}{\chi {D}_{laser}}.$$

Two limits are imposed in the OR experiment: (1) the power *P*_*THz*,*d*_ at the receiver must be larger than the noise equivalent power *NEP* and (2) the power density *D*_*laser*_ must be smaller than the sample material damage threshold *D*_*damage*_. Therefore, in this OR experiment, the laser beam can be focused to a minimum waist given by the following expression:

5 $${w}_{laser,min}\propto \mathop{\underbrace{\sqrt{\frac{NEP}{{{\rm{\Omega }}}_{lens}\,}}}}\limits_{{\rm{setup}}}\mathop{\underbrace{\frac{1}{\chi {D}_{damage}}}}\limits_{{\rm{sample}}}.$$

Because the THz light is generated in the sample area illuminated by the laser, the THz lateral resolution *δ*_*THz*_ is proportional to the minimum laser waist *w*_*laser*,*min*_. As expected, when *D*_*damage*_ is high, the laser beam can be strongly focused while keeping the laser power constant, resulting in a better THz resolution (*δ*_*THz*_ decreases). As well, using highly sensitive THz detectors (*NEP* small) like superconducting devices (hot electron bolometers, kinetic inductance detectors, etc.) may improve the THz signal detection threshold by 2 ~ 4 orders of magnitude^27^ and thus it would lead either to a sub-micron resolution, or to the study of fragile samples, like biologic tissues. However, because of the square root in Eq. (5), the resolution depends more on the sample damage threshold. Also, if the sample is highly nonlinear (large *χ*), the resolution is better. It follows from relation (5) that the spatial resolution depends on both the sample (*D*_*damage*_, *χ*) and the setup (*NEP*, Ω_*lens*_). In the present experiment, we observe damage in the sugar sample for ${D}_{laser} \sim 3.2\times {10}^{12}$ W/cm^2^ and we measure both the OR profile and the THz beam waist with ${D}_{laser} \sim 2.8\times {10}^{12}$ W/cm^2^. We selected this pump power density in order to have a large measurement dynamics and actually the generated THz signal (power) was about 1600 times larger than *NEP*. Therefore, the reported 50-*μ*m resolution could have been decreased by a factor $\sqrt{1600}=40$, i.e. down to a few *μ*m, at the expense of a strong lack of dynamics.

About the depth resolution of the technique for a bulk sample (*d* ≫ *λ*), we can simply estimate it from Gaussian optics. Typically, when using a focused pump beam, the nonlinear signal is generated in a material region whose thickness is equal to the Rayleigh length *Z*_*R*_ (${Z}_{R}=\pi {w}_{laser}^{2}{n}_{laser}/\lambda$)^28^, where *n*_*laser*_ is the refractive index of the nonlinear material. For thin samples, the depth resolution is the minimum among *Z*_*R*_ and the crystal thickness. In the present work, *Z*_*R*_ ~ 5 mm, therefore the depth resolution is equal to the crystal thickness. It is why the profile given in Fig. 5 right is directly proportional to the sugar grain thickness profile, assuming the material is homogeneous.

In conclusion, we have demonstrated that sub wavelength THz images can be obtained by OR in the sample. Here, while testing a sugar grain, we achieve a *λ*/12 lateral resolution. We also give an estimation of the limits of this technique, in terms of spatial resolution which is determined by both the sample (damage threshold and nonlinearity) and the setup (collecting optics and detector sensitivity).

## Methods

**Figure 6:**
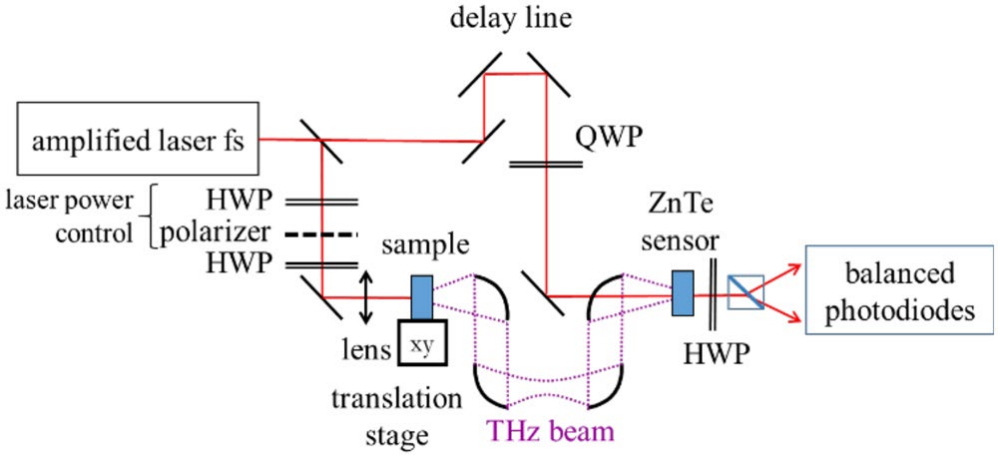
Experimental setup.

The studied grains are taken in a packet of commercial caster cane sugar (brown sugar). Caster cane sugar is made at 85–90% of sucrose and the rest (10–15%) includes fructose, glucose and impurities^29^. Sucrose belongs to the space group P21 (monoclinic)^30^. The size of the sugar grains is typically of the order of millimeters. We chose grains that exhibit as flat as possible upper and lower faces, which appear as if they have been cleaved. The studied sugar grains are stick on a transparent rubber tape. In the laser beam, the tape is located before the grain. Thus, the generated THz beam does not propagate through the tape. We have checked that the tape does not produce a noticeable THz signal when illuminated by the laser. The experimental THz set up is similar to a classical THz time-domain system, in which the emitting antenna has been substituted by the nonlinear sample, i.e. the sugar grain stick on the rubber tape (Fig. 6). The setup includes an amplified femtosecond laser system (Coherent Libra) that delivers 5 mJ pulses of 50 fs duration at a repetition rate of 1 kHz. The central wavelength of the laser pulses is *λ* = 800 nm. The power of the pumping beam is adjusted with a half-wave plate (HWP) and a polarizer. In front of the sample, a second HWP permits to rotate the laser polarization. The detection of the THz signal produced by the sample is recorded with an electro-optic antenna, which consists of a 200 *μ*m thick [111] ZnTe crystal, a HWP, a Wollaston prism and a balanced photodiode system. The parabolic mirrors used to collect the THz wave have a 2″ (~5.1 cm) diameter. The one close to the sample has a reflected focal length of 6″ (~15.2 cm) and the other ones 4″ (~10.2 cm). The THz signal was recorded with a lock-in amplifier (time constant: 1 s). We performed the measurements with spatial steps of 10 microns, taking in average 1 minute for each point. The total recording time of the whole image was around 3.5 hours.

Since all the sugar crystal axes directions are unknown, we changed the pump beam polarization angle by rotating the HWP situated in front of the sample, in order to maximize the detected signal. Then, we optimized the signal by rotating the HWP close to the balanced photodiodes. This allows to put the detection axis in the same direction of the THz electric field. Because the detecting ZnTe crystal is [111] cut, there is no need to rotate it to maximize the detected signal^31^.

## Data Availability

The datasets generated during and/or analysed during the current study are available from the corresponding author on reasonable request.
